# Cases of Pectoral Disease

**Published:** 1836

**Authors:** O. M. Herron

**Affiliations:** House Surgeon of the Commercial Hospital of Ohio


### Art. II.—Cases of Pectoral Disease. By O. M. Herron, M.D., *House Surgeon of the Commercial Hospital of Ohio.*

Samuel C. B., blacksmith, native of Ohio, aged 21, was admitted January 21, 1836, with a large abscess, on the right breast, a little below the nipple. He said that ten days previous to this he had suffered slightly from a pain in the region now occupied by the abscess. The tumor was large and occupied nearly the whole field of the pectoralis major muscle. Extreme tenderness along the inferior part of the neck on the right side, and from thence to the point of the shoulder. Nearly perfect paralysis of the right arm. Tongue very light and parboild in the centre and red at the margin; bowels constipated. Pulse 100: small, quick, cordid. Skin dry and feverish. The tumor was opened and discharged three pints of healthy looking pus, with immediate relief to the patient. A tight bandage and alterative medicines were used, and on the following day a weak solution of nitric acid was injected into the cavity of the abscess and repeated once a day. Quinan sul. gr. ij. every three hours.

January 29th.—Breast still suppurates considerably. The above treatment continued.

February 3d.—At irregular intervals he has paroxysms of coughing. The suppuration from the abscess is becoming extremely forbid. Towards evening the countenance is flushed, and the pulse wiry and quick. Tongue clean; bowels regular. Medicines continued. Adhesions of the parieties of the abscess have formed, so that the original dimensions are diminished to less than half. The whole region occupied by the abscess at first, is extremely tender. The costal cartileges are carious, but the piobe is not admitted into the cavity o the thorax. Slight cough, which produces great pain in the part effected. We injected the abscess with soap suds and water in order to clear the remaining cavity of all vitiated secretion, and *then* used the solution of nitric acid, or myrrh, as before.

23.—Cough much diminished. Great tenderness over the region of the lower part of the scaleni muscles, but no intumescence nor inflammation.

28.—The discharge from the abscess diminished considerably. Bowels rather lax, with evacuations of a thin and light yellow color. Tenderness at the origin of the scaleni muscles as great as ever. Oedema over the lower third of the sternum, with acute pain, upon pressure being made. Tongue slightly coated and somewhat hypertrophoid. The febrile hectic exacerbation visits him every evening, flushing the cheek with a crimson hue, which, in the morning, is pale. No night sweats. Pulse (at 5 P. M.) 102; quick, not *very* small, rather soft. ℞ hyd. sub. mer. gr. iij., ex. conii. macutali gr. ij. G. opii., gr. ¼ ter. die. Emplastrum cortes Peruvian.

April 10.—The part mentioned above, at the lower portion of the scaleni, has become tumefied; the puffiness passes over the clavicle and extends considerably below it. The intermescence is considerably increased when he takes the recumbent posture. This abscess, which did not communicate with the old one, discharged about a gill of bloody pus. Change of posture had no effect, either in promoting or diminishing the discharge from the abscess. The surface, at the part occupied by the old abscess, and which is not by the new, has ceased to be tender. All that part of the right thorax from the region of the first abscess to the top is greatly enlarged; the measure from the spine to the sternum on a line with the right mamma being two inches more than that of the left.

April 15.—The tenderness over the whole region is much diminished. Cough slight. Tongue clean. Skin moist. Tonics and wine. Moxa along the right side of the spine. The moxa produced pretty good ulcers, which were kept open for some time. The injections, wine and tonics, are continued. The discharge from the abscess gradually diminished, and his general health slowly recruited, until he was considered able to travel by the river and canal to his father's who had long been anxious for his return home. At the time of leaving, he was considerably emaciated; skin fair and transparent; conjunctiva of pearly whiteness. Tongue clear, and rather indicative of chronic gastritis. No improvement in the shape of the thorax. No expectoration since his admission.

Case 2.—John C., a butcher, a native of Germany, and aged 24, was admitted 27th May, with a running abscess in the right breast; the orifice was on the lower part of the mamma. He said, that nine weeks before his admission he had a slight attack of common catarrh, but that this soon passed off. That about this time a tumor commenced upon his right breast, unattended by any pain or soreness whatever,—even when pressure was made upon it:—that this gradually increased for several weeks, was then opened and discharged about a tea-cupful of matter. That there had not been, from the commencement, the least redness, nor any other discoloration of the skin. The orifice had kept open and a considerable discharge had continued up to the time of his admission. He is not as yet (24th June) much emaciated; on the contrary, rather full and muscular. Features of the face obtuse. Complexion sallow. Tongue tumid and light colored. The same treatment was pursued as in the last mentioned case. He was not afflicted with cough at any time after his admission. For a short time his bowels were somewhat constipated, but alteratives of hydrag. massan cum. pulv. ipecacuanha compos., soon corrected this. A short time previous to the termination of the case, a diarrhœa, supervened, which, however, was checked with the treatment ordinarily pursued in such cases. At his first entering the hospital, the fœtor from the purulent discharge was considerable, and increased so that it became necessary to remove him to a private room. A free opening was early made, for the discharge of matter, and the cartilages were found denuded and ulcerated. For a day or two at a time, the discharge would almost cease, but then return again as bad as ever. These alternations continued without the supervention of any other appearances worthy of notice until he died.

The thorax and abdomen were opened in the presence of several medical students. The right cavity of the thorax contained nearly three pints of what is called healthy looking pus. The costal cartilages were ulcerated (as was observed before) in three different places, and there were two openings from the cavity of the thorax into that of the abscess: one about the size of a six cent piece, and the other about one-third that size. The ulcerations of the cartilages were upon the side next the abscess, (external,) and were covered by a black secretion. There was not a vestage of pulmonary tissue in the right cavity of the thorax, and the pleura, which was greatly thickened, was coated internally by a spongy membrane, covered by numerous papillar upon the thoracic side. This adventitious membrane covered the stumps of the bronchial tubes—not a particle of those tubes even penetrated the cavity—and rendered them impervious. The upper lobe of the left lung was atrophoid and corrugated.

The inferior one healthy. The pericardium contained about three ounces of yellowish serum. The heart was a little hypertrophied, of a light straw color, and exceedingly softened, so much so as to be easily torn with the fingers; the parieties were of the ordinary thickness. The abdominal viscera, including the mucus membrane of the alimentary canal presented no diseased appearances of recent date. The color had traces of an old diarrhœa.

Case 3.—Custis was admitted on the —day of June, 1836. He is a native of Germany, was raised until a few years past on the banks of the river Rhine. Before leaving his native country, he suffered from ascites. At the time of his admission there was no prominent ailment of a serious or painful character. General debility induced the necessity of his coming into the house. He is rather tall and masculine, hair light, eyes grey, conjunctiva pearly, forehead prominent, skin transparent, complexion a light sallow. Considerable enfiltration of the cellular tissue beneath the chin, so as to form a sack somewhat pendulous. The cellular tissue over the anterior part of the abdomen and chest is also considerably distended with what appears, more than any thing else, like their adipose matter. The cuticle of the abdomen has that cicatrised and corrugated appearance so remarkable in females who have borne many children and neglected to maintain a tight bandage for a sufficient time afterwards. There is immense distention of the cellular tissue over the abdomen. Upon making considerable pressure, a trifling tenderness was found over the cartilage of the sixth rib. Tongue rather light colored, a little tumid, pulse natural, skin moist enough. A few hydragogue cathartics were given at first, but found inadmissible on account of the too long continued irritation of the alimentary canal, which they reduced, after resorting to several diuretics and diaphoroetics without benefit, he was directed to take the solution of hydriodate of potash. In about ten days a slight elevation commenced over the fifth, sixth, and seventh costal cartilages, and near the sternum. In six or seven days, this had become a considerable tumor, and when opened, discharged a sanious and floculent pus. Upon the first appearance of this elevation, frigorific applications were made, but failed to repress it. After its contents were discharged it disappeared, and the orifice healed kindly. In about a fortnight another intumescence, precisely similar, made its appearance, a little to the right of the first, and occupying a part of the sternum—in both, the skin continued perfectly unchanged in color, and scarcely any pain was produced, even when pressure was made upon the part. Over the last one a constant irritation has been kept up on the skin, with Brodie's liniment, and it has vanished. The patient has not been troubled with cough at any time since his admission.

Post mortem of case 4. July 25.

Brig. C., age 26, laborer, died yesterday. He was admitted, in the first place, about two months ago, with some general debility, moderate diarrhœa and cough, attended with pain in the right breast. By the use of the ordinary internal and external remedies, his general health was improved and the distress in his breast greatly mitigated. But being anxious to visit his friends, who reside at no great distance, he was permitted to depart. About a week ago he returned with the symptoms of pectoral disease, aggravated in a high degree. Tongue coated with yellow fur; cough severe, with pain in the right side. Breath excessively fœtid, and the expectoration equally so. Stomach irritable. Considerably emaciated. Eyes sunken and conjunctiva white. The expectoration was extremely profuse in the mornings.

Inspection. Left lung nearly healthy; some cicatrices at the apex, but no tubercles. Right lung extensively adherent to the costal pleura, with an immense phagedenic abscess occupying nearly the whole of the superior lobe, and containing a small quantity of dark greenish pus, of the most offensive odor imaginable. The cyst of the abscess was irregular and ragged and the contiguous lung indurated, and of a dark red hue. Heart pale, hypertrophied, and *greatly softened*. Liver somewhat enlarged and blanched, inferior surface of the lobes extremely black. Spleen.—Convex surface mottled, a little hypertrophied, and the inferior half of a redish purple color; the discoloration, from a very light blue, commencing abruptly at a transverse line near the centre. Omentum a filament. Glands of the messentary undurated and slightly enlarged. Gall bladder atrophied, and containing about two drachms of light colored bile. Bowels.—The vessels were considerably injected along the jegunum and ilium, and the color at two or three places greatly constringed. The mucus membrane of the stomach presented no appreciable lesion, in the duodenum, the glands of Brunner (glandula solitaria) appeared healthy. In the ilium, the mucus membrane was softened, the glands unulcerated. There was ulceration of the mucus membrane of the colon at the constringed points.

